# ‘I Can’t Concentrate’: A Feasibility Study with Young Refugees in Sweden on Developing Science-Driven Interventions for Intrusive Memories Related to Trauma

**DOI:** 10.1017/S135246581600062X

**Published:** 2017-03-01

**Authors:** Emily A. Holmes, Ata Ghaderi, Ellinor Eriksson, Klara Olofsdotter Lauri, Olivia M. Kukacka, Maya Mamish, Ella L. James, Renée M. Visser

**Affiliations:** 1 Department of Clinical Neuroscience, Karolinska Institutet, Sweden; 2 Medical Research Council Cognition and Brain Sciences Unit, Cambridge, UK; 3 Forced Migration Trauma Service, Central and North West London NHS Foundation Trust, London, UK; 4 London School of Economics, Department of Psychological and Behavioural Sciences, London, UK

**Keywords:** Refugees, psychological trauma, intrusive memories, concentration

## Abstract

**Background:**

The number of refugees is the highest ever worldwide. Many have experienced trauma in home countries or on their escape which has mental health sequelae. Intrusive memories comprise distressing scenes of trauma which spring to mind unbidden. Development of novel scalable psychological interventions is needed urgently.

**Aims:**

We propose that brief cognitive science-driven interventions should be developed which pinpoint a focal symptom alongside a means to monitor it using behavioural techniques. The aim of the current study was to assess the feasibility and acceptability of the methodology required to develop such an intervention.

**Method:**

In this study we recruited 22 refugees (16–25 years), predominantly from Syria and residing in Sweden. Participants were asked to monitor the frequency of intrusive memories of trauma using a daily diary; rate intrusions and concentration; and complete a 1-session behavioural intervention involving Tetris game-play via smartphone.

**Results:**

Frequency of intrusive memories was high, and associated with high levels of distress and impaired concentration. Levels of engagement with study procedures were highly promising.

**Conclusions:**

The current work opens the way for developing novel cognitive behavioural approaches for traumatized refugees that are mechanistically derived, freely available and internationally scalable.

## Introduction

The global refugee crisis is escalating, as exemplified by events in Syria (United Nations High Commissioner for Refugees [UNHCR], 2015). Even once in a safe country, refugees are often plagued by vivid mental images of traumatic events – called ‘*intrusive memories’* – that repeatedly spring to mind unbidden (American Psychiatric Association, 2013). Exposure to traumatic events, within Syria or on the escape to Europe, have profound mental health consequences (Hassan et al., 2015; Abbott, 2016; Nature Editorial, 2016) that interfere with acquiring skills needed to integrate into the new environment (e.g. language learning), for example by disrupting concentration. The UNHCR report on the mental health of Syrians (Hassan et al., 2015) highlights culture-specific idioms of distress such as ‘*mou adder rakkezz’* – ‘I can’t focus, I can’t concentrate’. Intrusive sensory memories occur across several psychiatric disorders (Bryant et al., 2011), notably in those disorders with high prevalence in refugees (e.g. Bogic et al., 2012; Hollander et al., 2016). These include post-traumatic stress disorder (PTSD) (Grey and Holmes, 2008), depression (Birrer et al., 2007; Williams and Moulds, 2008) prolonged grief (Bryant et al., 2014), and psychosis (Morrison, 2004). Intrusive sensory memories present a possible ‘transdiagnostic’ target for intervention (Fairburn et al., 2003).

Of the more than a million refugees that reached the European Union in 2015 (UNHCR, 2015), Sweden has taken the highest numbers per capita with 162,877 asylum applications in 2015 alone (The Swedish Migration Agency [Migrationsverket], 2015). Almost two-thirds of these applications were from individuals aged 13–24 years, and many from unaccompanied minors, constituting 50.3% of all children between 0 and 17 years who applied for asylum in Sweden in 2015 (The Swedish Migration Agency [Migrationsverket], 2015).

Trauma-associated mental health problems incur heavy costs to individuals and society: there is urgent need for language-free, simple-to-deliver treatments that can reach a large and dispersed refugee population (Ullmann et al., 2015). However, innovation is sorely needed as most psychological treatments cannot reach people with mental illnesses at international scale (Kazdin and Blase, 2011; Kazdin and Rabbitt, 2013). Refugee host countries lack capacity to deploy evidence-based psychological treatments at the scale needed as they typically require face-to-face contact with a therapist/lay worker (Patel et al., 2011) alongside interpreters (Ullmann et al., 2015). Furthermore, the number of psychological treatments tested with refugee populations is low (Nickerson et al., 2011), raising questions of cultural acceptability (Hassan et al., 2015).

Approaches could be: (i) to utilize smartphone technology – which is appealing and accessible to young people from Syria (87%; Mohr et al., 2014; Central Intelligence Agency [CIA], 2016) – in a given language, e.g. Arabic; (ii) to take a mechanistically focused cognitive approach from experimental psychopathology and target one tractable symptom (rather than a whole syndrome); and (iii) to focus not only on reducing psychological distress but also downstream improvements in *functioning* (e.g. adaption to a host country), particularly given refugees’ motivation for improvement, demonstrated by travelling miles through danger and obstacles to reach a better life.

We propose that certain behavioural interventions could be delivered at minimal cost, directly (without a therapist/interpreter), by smartphone, in refugees’ daily life. Our vision is galvanized by the recent concept of ‘massive open online interventions’ (MOOIs) (Muñoz et al., 2015). ‘*MOOIs have the potential to increase the reach, scalability, and affordability of psychological interventions … This term was inspired by the growing popularity of massive open online courses* (*MOOCs*) *… which attract millions of students …. Digital interventions … can be used again and again by people anywhere in the world*’ (Muñoz et al., 2015).

We have experimentally derived simple-to-deliver methods to reduce the frequency of intrusive memories of trauma via computer game-play. A one-session behavioural intervention (involving the game Tetris) at specified time points, reduces the frequency of intrusive memories of experimental trauma (film footage) by approximately half (Holmes et al., 2009; Holmes et al., 2010; James et al., 2015). This suggests a technique that could be developed after real trauma. Mental disorders and attending mental health services is associated with stigma in Syrian cultures (Hassan et al., 2015). Young people particularly can be reluctant to access psychiatric/psychological services, thus computer-gaming in daily life opens new possibilities to provide support in non-/less-stigmatizing ways.

This feasibility study with young refugees recently residing in Sweden is a first step towards future scalable intervention development. We explored monitoring one target symptom, simple measures of functioning, and the acceptability of a novel intervention procedure. Participants were asked to (a) monitor the frequency count of intrusive memories of trauma for one week using a daily intrusion diary; (b) rate concentration disruption and intrusive memory occurrence over a week; and (c) complete a one-session intervention involving Tetris gameplay via smartphone. An important aspect of the research was whether these procedures were feasible without an interpreter, given that reliance on interpreters would massively increase treatment costs and impede scalability.

## Method

### Participants and recruitment procedure

Twenty-two young refugees (mean age 22 years, SD = 2.4; 17 males, 5 females) participated. Sixteen (73%) were from Syria, three from Iraq, and three not specified. Twenty participants (91%) had Arabic as their first language; the remaining two (9%) spoke Arabic, but had a different mother tongue. Eighteen participants (82%) had arrived in Sweden in the last 6 months before study participation; three individuals arrived 7–12 months earlier, and one individual arrived more than 12 months earlier. Ten participants (46%) had had more than 12 years of education, seven participants (32%) had between 10 and 12 years, four participants (18%) had between 6 and 9 years, and one participant had less than 5 years of education.

Participants were recruited via posters in Swedish, English and Arabic, advertised digitally through social media (e.g. Facebook pages of Refugees Welcome and Save the Children) and advertised in paper in relevant institutions (non-governmental organizations [NGOs], religious institutions, cultural initiatives, government agencies, and refugee accommodation), and by handing information sheets to passing individuals at refugee accommodation (with help from local staff members). Sixteen people were recruited via refugee accommodation (73%), two via Swedish language classes (9%), two via direct approach at the central station in Stockholm (9%), one (5%) via Facebook, and one (5%) via a NGO. Recruitment and interviewing was done in Swedish/English (orally and on paper) and in Arabic (on paper), without the presence of an interpreter. Afterwards, the participant’s written responses were translated from Arabic/Swedish into English.

Participants did not receive any financial compensation. All gave their written, informed consent before participation; in case of minors consent was given in agreement with their custodian. The authors assert that all procedures contributing to this work comply with the ethical standards of Regional Ethical Review Board Stockholm on human experimentation and with the Helsinki Declaration of 1975, and its most recent revision (ethics approval number 2015/1915-31/5).

### Apparatus and materials

#### Intrusive memory monitoring via daily intrusion diary

**Figure 1:**
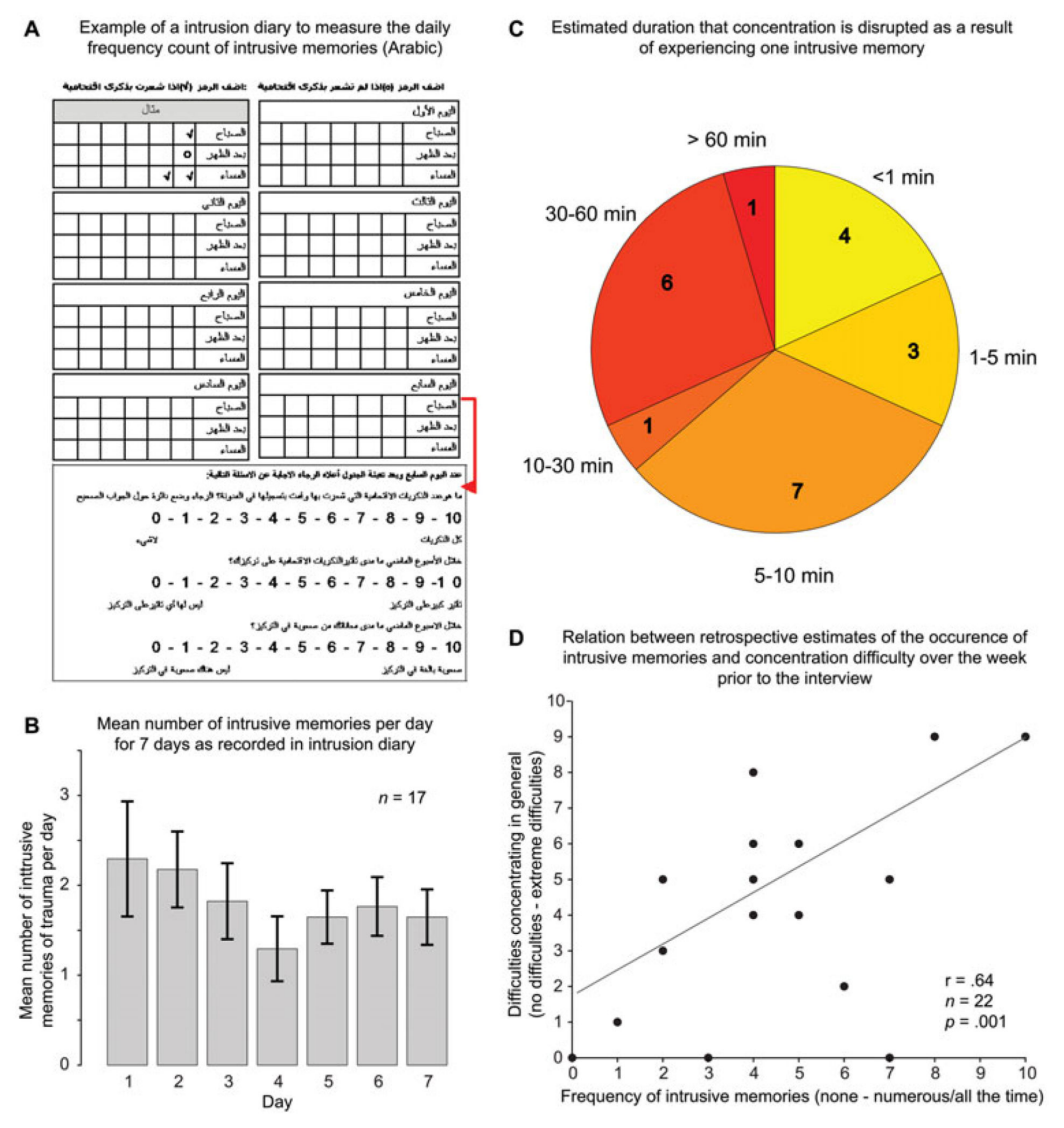
Example of paper-and-pencil intrusion diary in Arabic, used to assess the frequency count of intrusive memories of trauma daily for 1-week. Participants indicate with ticks per day how many intrusive memories they had in the morning, afternoon and evening, and to write down a zero if they did not experience any intrusive memories (A). Bar graph depicting number of intrusive memories of trauma in the intrusion diary per day for one week (returned by 17 participants), indicating a mean of almost two intrusive memories a day (M = 1.81, SD = 1.39). The mean count over the week was 12.65 (SD = 9.71). Error bars depict standard error of the mean (B). Pie chart displaying the estimated amount of time that a single intrusive memory disrupts concentration. Part size represents number of participants reporting a given duration (C). Scatterplot showing a relation between the occurrence of intrusive memories in the preceding week measured on as scale from 0 (none) to 10 (numerous/ all the time), and general concentration difficulties measured on a scale from 0 (no difficulties) to 10 (extreme difficulties) (D)

The total number (count data) of intrusive memories in the week after the interview was assessed using a daily pen-and-paper intrusion diary (Fig. 1A) in everyday life, as in previous experimental studies (Holmes et al., 2009; Holmes et al., 2010; James et al., 2015) and clinical studies (Iyadurai and Holmes, 2015; Horsch and Holmes, 2016). Participants ticked a box for the day and time period (morning/afternoon/evening) when the intrusive memory occurred, or marked ‘zero’ if they experienced none. Intrusive memories were described as: ‘images of traumatic events that pop into your mind without warning. They often take the form of visual pictures in your mind’s eye, e.g. like a snapshot image or a film clip. They can also include other senses such as sounds and smells, such as the smell of smoke. They may or may not be triggered by something you are aware of, such as telling someone about what happened, or watching something on the news.’ Participants were not to record memories recalled deliberately or general verbal thoughts. Instructions were based on previous protocols (see Supplemental Material) and a short definition of flashback/intrusive memories was printed on the intrusion diary as a reminder. Check marks were summed to yield daily and weekly frequency counts (Fig. 1B).

After 7 days, participants rated ‘How many of your flashbacks do you think you recorded in the diary’ from 0 (none of them) to 10 (all of them). Participants were given a stamped addressed envelope to return the intrusion diary by post.

#### Ratings of concentration disruption

Participants were asked to estimate, ‘When you have an intrusive memory how long does it disrupt your concentration on average?’, on an ordinal scale depicting different time intervals (<1 min, 1–5 min, 5–10 min, 10–30 min, 30–60 min, >60 min; Fig. 1C).

Two questions rated on 11-point visual analogue scales were: ‘Over the past week, how much difficulty did you have concentrating generally?’ from 0 (no concentration difficulties) to 10 (extreme concentration difficulties) (Fig. 1D); and ‘Over the last week, how much did your intrusive memories/flashbacks disrupt your concentration?’ from 0 (not at all disruptive) to 10 (extremely disruptive).

#### Rating of intrusive memory re-occurrence over the week preceding interview

Participants were asked ‘How many intrusive memories did you have in the last week?’ anchored from 0 (none) to 10 (numerous – all the time) (Fig. 1D).

#### Computer game-play and intervention procedure

Participants downloaded and installed the computer game Tetris to their smartphone (e.g. Tetris Free via https://play.google.com/store/apps; version 1.8.03.5593836126994432, developed by EA Mobile Montreal Team). Instructions for game-play were based on previous protocols (Iyadurai and Holmes, 2015); see Supplemental Material.

The cognitive task intervention procedure involved two key components: thinking back to a traumatic event followed by playing the computer-game Tetris (Iyadurai and Holmes, 2015; James et al., 2015; Horsch and Holmes, 2016). The following instructions were used to reactivate trauma memory: ‘We asked you in the information sheet about any traumatic events that you may have experienced. You don’t need to tell about this, but to make the computer game as helpful as possible, we just need to make sure that the traumatic event is in your mind just before you do the task. So I’d like you to think back to the traumatic event for a brief moment, specifically the worst moments that pop back to mind (intrusive memories that flash to mind). You don’t need to think about it in detail. Let me help you – did anything pop into your mind? Are there any images that are flashing to mind or any bits of the traumatic event you can see again? It’s OK, you don’t have to tell me what happened, as long as the images were in your mind that’s fine.’ Following instructions and a practice, participants were asked to play Tetris for at least one uninterrupted period of 15 min (or longer if they wished) on their smartphone.

#### Feasibility and acceptability

Feedback on the feasibility and acceptability of symptom monitoring and the potential for smartphone-delivered interventions was obtained using ratings scales as well as open-ended questions. For example, acceptability of the intervention was assessed by the following 11-point scale: ‘Do you think computer game play would be an acceptable way to reduce the daily frequency of the intrusive memories?’ from 0 (not at all acceptable) to 10 (very acceptable). An example of an open-ended question related to preference for mode of delivery of the intervention was ‘How would you feel about talking to a doctor/psychologist about your traumatic experiences?’

### Procedure

All study documents contained both English and Arabic text interspersed on the same sheets. Communication was done mostly through the translated texts (the interviewers did not speak Arabic) by handing them in the correct order to the participants for them to read and fill out.

Participants completed concentration and intrusive memory ratings, followed by the computer game-play cognitive task procedure. This involved reactivating a memory of trauma and 15–20 min of Tetris game-play on their smartphones. Instructions for the daily intrusion diary were given. Next, feasibility and acceptability feedback questions were collected (see Supplemental Material), with answers written by the participant in their preferred language. The session lasted between 60 and 90 min.

Participants were instructed to return their intrusion diary in the post after 1 week.

### Data analysis

Twenty-two participants were tested (Julious, 2005). The study aimed to provide estimates of the mean and variance of the number of intrusive memories and concentration difficulties people experience, to inform future studies, but not to conduct tests of statistical significance. However, given a directional hypothesis, the relation between the occurrence of intrusive memories and concentration difficulties was calculated using a Pearson correlation coefficient.

## Results

### Intrusive memory monitoring via intrusion diary

All 22 participants indicated that they were willing to fill out a daily intrusion diary to measure the frequency of their intrusive memories of trauma (7–10 on a scale from 0 to 10). Seventeen participants (77%) then returned the pen-and-paper intrusion diary by post after 1 week. Of the five that did not return the diary, one felt too sad to complete the study due to receiving bad news from Syria about family (as we were informed by his custodian), and one appeared to not have understood the information properly. The other three dropped out without providing a reason and did not reply to mobile phone SMS (short messaging service).

Critically, diary data indicated a mean of almost two intrusive memories per day (mean = 1.81, SD = 1.39). The mean number of intrusive memories weekly was 12.65 (SD = 9.71). Of the 17 participants who returned the intrusion diary, 13 (59%) rated 5 or higher on a scale from 0 (none) to 10 (all of them) when asked how many of their intrusive memories they had recorded in their diary (mean = 6.53; median = 6.00; mode = 6; SD = 2.62; range 2–10).

### Ratings of concentration disruption

Eighteen participants (82%) indicated that a single intrusion disrupts their concentration for longer than a minute on average, a majority (68%) indicated that it disrupts their concentration for longer than 5 min, while almost a third (32%) indicated that it disrupts their concentration for longer than 30 min (Fig. 1C). Difficulties in concentrating (in general) were experienced by most participants (Table 1), with 41% of the participants rating 7–10, on a scale from 0 to 10. When asked how disruptive intrusive memories are for concentration on a scale from 0 to 10, almost one-third of the participants (27%) indicated 5–7, and over a third of the participants (36%) indicated 8–10 (Table 1).

#### Rating of intrusive memory occurrence over the week prior to feasibility study

On a scale from 0 (none) to 10 (numerous), 21/22 participants rated that they had experienced intrusive memories of trauma in the past week, with almost a third of the participants (27%) rating 8–10. Only one of the 22 participants indicated that they had not experienced any intrusive memories over the preceding week.

There was a significant positive correlation between the degree to which participants had experienced intrusive memories over the preceding week and the degree to which they experienced concentration difficulties (*r* = 0.64, *n* = 22, *P* = 0.001; Fig. 1D).

#### Feasibility and acceptability of smartphone-delivered interventions

All participants had access to a smartphone and were able to download the Tetris app. Ratings indicated that it was very easy to download the app (Table 1). The majority of participants (59%) reported that they were not familiar with Tetris prior to the study.

A majority of the participants (77%) rated 5–10 on a scale from 1 to 10 when asked whether they would find it useful to have fewer intrusive memories each day (Table 1).

All participants completed at least 15 min of game-play (mean = 18 min; SD = 2.4). Ratings suggested that most participants found Tetris game-play easy and enjoyable and that it distracted them from having unpleasant thoughts, images or feelings (Table 1). Furthermore, ratings suggested that they would likely play Tetris by themselves, in a different environment and that they would recommend it to a friend (Table 1).

Finally, a majority of the participants believed a computer game would be an acceptable way to reduce the daily frequency of intrusive memories (Table 1). Preference for doctor/psychologist *versus* technology was equivocal (Table 1). When asked ‘Why?’ replies included: ‘Because I can talk to the doctor and this will give me the feeling that he will understand my problems better’; ‘I like computer games, they are fun and make me feel good and happy. Playing the game makes me forget about my burdens’; ‘It is suitable because we rely on technology in almost everything. Smart phones are accessible and always nearby’; ‘Because it’s more easy and the phone now is my best friend to us’.

## Discussion

In this study we explored intrusive memories of trauma and concentration in young refugees predominantly from Syria who recently arrived in Sweden. Specifically, we assessed the feasibility and acceptability of methodology required to develop science-driven interventions aimed at reducing the frequency of intrusive memories of trauma. Levels of engagement with study procedures were highly promising. Results suggest a high prevalence of intrusive memories and concentration difficulties in this population and suggest a relationship between these symptoms: more than half of participants indicated that each intrusive memory disrupted their concentration for more than 5 min. Furthermore, participants reporting a higher frequency of intrusive memories also reported more concentration problems in general. Free report data suggested related functional impairment on tasks associated with adaptation to the host country such as language learning.

Supporting the development of novel and scalable psychological intervention procedures (Kazdin and Blase, 2011; Kazdin and Rabbitt, 2013), our data indicate that young refugees are able and willing to (a) monitor a target symptom – the frequency count of intrusive memories of trauma for 1 week using a daily intrusion diary; (b) rate intrusive memories and concentration levels; and (c) complete one session of computer game-play (Tetris) on their smartphone as part of a mechanistically driven intervention procedure. These procedures were feasible without an interpreter, highlighting the potential large-scale application of such interventions in line with the concept of a future MOOI (Muñoz et al., 2015).

We have argued that psychological treatments can benefit from advances in neuroscience (Holmes et al., 2014). Candidate mechanisms to alter trauma memory are offered by understanding the neuroscience of memory reconsolidation (Nader and Einarsson, 2010), alongside insights from cognitive psychology (Andrade et al., 1997) which capitalize on special features of intrusive memories – disrupting their mental imagery-based nature (Grey and Holmes, 2008; Engelhard et al., 2011; Pearson et al., 2015). Earlier experimental work shows that the effect of the cognitive-task intervention procedure in reducing intrusive memory frequency is specific to visuospatial tasks (e.g. Tetris) when compared with a verbal control game (Holmes et al., 2010). Further, this method is effective not only for recently viewed trauma footage, but also for established memories (James et al., 2015). A recent randomized control trial (Iyadurai and Holmes, 2015) has successfully extended findings to a hospital emergency department setting with patients after a traumatic road traffic accident. A main advantage of this technological intervention, as underscored by results from the current study, is the potential for therapist-free delivery, enabling large-scale dissemination (for example via a MOOI; Muñoz et al., 2015) and cultural acceptability to young refugees.

Limitations of this feasibility study include the small sample size and the sampling procedure, which may have resulted in a less representative sample, and possibly an underestimation of the scale of the problem (individuals with more severe psychological problems may be reluctant to volunteer for research studies). Further limitations include the lack of a control group and a full clinical interview to assess post-traumatic symptoms. Instead, we took a pragmatic approach, given the urgency of the topic. Whilst the study is modest, alongside the paucity of research in this area and the current ‘epidemic’ scale of traumatized refugees, results offer a small step towards scalable innovation: new ways to (a) develop and deliver psychological treatments for refugees by focusing on one target symptom (not a syndrome); (b) deliver research materials in another language; and (c) bridge a novel behavioural treatment approach for mental health with human memory theory.

Compliance with the 1-week intrusion measurement was high (78% of participants returned the diary by post). The 22% drop-out is remarkably low given that most participants were not familiar with the concept of paper postal services. Following the impact of war there has been no postal service in Syria for some years, with smartphone technology almost entirely replacing its use. We included a photograph of a post-box in the study material to explain the concept of paper diary return. Future studies harnessing technology for symptom monitoring would probably improve compliance (Malik et al., 2014). This is corroborated by the finding that attitudes towards using smartphone technology for symptom monitoring and intervention delivery were generally positive.

Participants reported, on average, two intrusions a day, which is higher than the maximum score (‘daily’) on the Clinician-Administered PTSD Scale for DSM-5 (CAPS-5) (Elhai et al., 2005). A goal to reduce one intrusion per day could represent a change from CAPS-5 maximum (‘daily’) to mid-minimum (‘once-or-twice a week’/‘never’), representing a clinically meaningful outcome target for future research.

Participants indicated that their intrusive memories typically disrupted their concentration for several minutes (mode 5–10), suggesting that intrusive memories were not only distressing, but also impaired functioning by disrupting focus on daily tasks (see Supplemental Material). Given that persistent intrusive memories are common after trauma, even in the absence of full clinical criteria for PTSD, intrusive memory and their associated ‘hotspots’ in the underlying trauma memory constitute an important target for treatment (Grey et al., 2002). Reducing intrusions holds promise to *prevent* the deterioration of mental health, and further to promote improvements in daily functioning, such as language-learning, key to integration for refugees entering a new country (Abbott, 2016; Nature Editorial, 2016), and associated with later employment (Daniel and Zurawan, 2010). Behavioural interventions focusing on a tractable target symptom (here intrusions) may have knock-on effects on other risk factors, promoting resilience and adaptive functioning. Scalable psychological treatments have long been needed (Kazdin and Blase, 2011; Kazdin and Rabbitt, 2013) and the current refugee crisis makes their development an imperative direction for clinical psychological science.

## Supplementary Material

To view supplementary material for this article, please visit https://doi.org/10.1017/S135246581600062X

Supplementary Material

## Figures and Tables

**Table 1:** Concentration and the feasibility and acceptability of a smartphone game-play intervention

Rating scale	*n*	Mean	Median	Mode	SD	Range
**Concentration**						
Over the past week, how much did your intrusive memories disrupt your concentration?^a^	22	5.73	6.00	10	3.68	0–10
Over the past week, how much difficulty did you have concentrating generally?^b^	22	5.68	5.50	9	3.59	0–10
**Feasibility and acceptability**						
*Smartphone-delivered interventions*						
Would you find it useful to have fewer intrusive memories of trauma each day?^c^	22	6.55	6.50	10	2.77	1–10
How easy was it to download the app?^d^	21	9.67	10.00	10	1.32	4–10
How easy did you find playing Tetris?^e^	22	6.73	6.00	5	2.19	3–10
Did you enjoy playing Tetris?^f^	22	7.55	9.00	10	3.33	0–10
Did you feel that Tetris distracted you from having any unpleasant thoughts/images/feelings?^g^	22	7.45	8.00	10	2.26	2–10
How likely is it that you would play Tetris by yourself, in a different environment?^h^	22	6.77	8.00	10	3.38	0–10
Would you recommend playing Tetris to a friend?^i^	22	8.36	9.50	10	2.40	1–10
Do you think computer game-play would be an acceptable way to reduce the daily frequency of the intrusive memories?^j^	20	6.65	7.00	10	2.94	0–10
How much would you prefer an intervention that is delivered by a computer/smartphone compared to seeing a doctor/psychologist in person?^k^	21	5.62	6.00	10	3.75	0–10

a 0 = not at all disruptive; 10 = extremely disruptive;

b 0 = no concentration difficulties; 10 = extreme concentration difficulties;

c 0 = not at all useful; 10 = very useful;

d 0 = not at all; 10 = very easy;

e 0 = not at all; 10 = extremely easy;

f 0 = not at all; 10 = very much enjoy;

g 0 = not at all; 10 = very much;

h 0 = not at all; 10 = very likely to play;

i 0 = certainly not; 10 = certainly recommend;

j 0 = not at all acceptable; 10 = very acceptable;

k 0 = prefer doctor/psychologist; 10 = prefer smartphone/computer; SD, standard deviation.
