# Supplementary material for: ‘I Can’t Concentrate’: A Feasibility Study with Young Refugees in Sweden on Developing Science-Driven Interventions for Intrusive Memories Related to Trauma
Source: Behav Cogn Psychother. Author manuscript; Available in PMC 2019 Jul 1. (PMC6600797; doi:10.1017/S135246581600062X)
Supplement: Supplementary Material [file EMS83497-supplement-Supplementary_Material.docx]

**“I can’t concentrate”: A pilot study with young refugees in Sweden on intrusive memories related to trauma**

Emily A. Holmes, Ata Ghaderi, Ellinor Eriksson, Klara O. Lauri, Olivia M. Kukacka, Maya Mamish, Ella L. James, Renée M. Visser

**Supplemental Material**

**Additional procedures**

**Intrusive memory diary instructions.** “So here it summarises what we mean by an ‘intrusive memory’. In translating this document from English to Arabic, it was hard to find the right word to describe intrusive memories. In Arabic, there are different meanings. There are brief images of traumatic events that pop into your mind without warning (often visual pictures); there is fully reliving the traumatic event and acting or feeling as if it was happening again; and there are nightmares of the traumatic event. Here, we are particularly interested in brief images or pictures that pop into your mind, which are the most common. However, we are also interested in fully reliving the event and nightmares of the event, which tend to be less common.

So to explain a bit more, intrusive memories are images of traumatic events that pop into your mind without warning. They often take the form of visual pictures in your mind’s eye e.g. like a snapshot image or a film clip. They can also include other senses such as sounds and smells, such as the smell of smoke. They may or may not be triggered by something you are aware of, such as telling someone about what happened, or watching something on the news.

Intrusive memories of traumatic events can vary from being vivid and emotional to being very short, fleeting and broken up. Does that make sense, what I mean by an intrusive memory? And just to make it clear, intrusive memories are not the same as deliberately thinking about a traumatic event or mulling it over. They are also not the same as general thoughts about the event without an image, such as thinking “I had a bad experience”. Does that seem clear?”

**Instructions for Tetris game-play.** First, participants, and their family if they were present, were introduced to the idea that doing something constructive, like playing a visual computer game such as Tetris (e.g., TETRIS FREE via https://play.google.com/store/apps; version 1.8.03.5593836126994432 developed by EA Mobile Montreal Team), may help with the upsetting and intrusive memories that people sometimes experience after traumatic events, with the overall aim of this study being to help develop new treatments to support people in the future. Participants were advised that they would be asked to think back to a traumatic event for a brief moment and then play the computer game for about 10-20 min without being disrupted.

Before memory recall, participants practiced the game (in ‘Marathon mode’) for a few min with the researcher. Particular attention was given to asking participants to play Tetris in a way that encouraged mental rotation and brought out the colour and spatial properties of the game. During game-play participants received encouragement and practical advice when needed to maintain attention and focus on the game. They were reassured that while it is good to keep playing the game without interruption, the actual score is not important, rather to enjoy game play.

**Additional results: answers to open-ended questions**

**Intrusive memories and concentration disruption.** Answers to open-ended questions about how intrusive memories of trauma interfere with concentration included: “[intrusive memories] makes me lose my concentration”; “Sometimes, the past events cross my mind, I start thinking about them. This makes me feel sad and unable to concentrate anymore.”; “In school I understand nothing about what the teachers are talking about when I get sad. Then I go home and I want to sleep.”; “[intrusive memories] have negative impact. They occupy the mind […]”; “When I go back to the memories of the past, I become very distressed. I try to change location to avoid the severe psychological pain occurring after recalling these memories”.

Intrusive memories of trauma were reported to interfere with their ability to learning new skills, such as language, e.g., “[intrusive memories] have a bad impact on my ability to concentrate and on remembering new words”; “Sometimes, my concentration gets disrupted by the intrusive memories, this doesn`t allow me to practice new skills like learning the language for instance.”; “When I experience the intrusive memories I can`t focus on anything, not learn anything new”; “It has a great impact. For instance, when trying to learn the language, I can`t spend more than two hours reading. I leave the book after that because I can`t continue.” Some answers reflected a more pervasive impact on daily functioning, e.g., “All the time I forget what I am doing. I go into a room and do not remember why. I leave things and don’t remember where. My phone is often lost, even when it is in my hand sometimes.”

**Acceptability of smartphone delivered interventions versus face-to-face interventions.** The majority reported positive attitudes toward an intervention involving playing a computer game to help reduce intrusive memories, for example “Good initiative, I hope it will be useful.”; “I think it is an amazing way.”; “Excellent idea, very good.”. Some participants were neutral, e.g., ‘I don’t know. Maybe it can help’. Two participants were clearly sceptical: “I don’t agree on using computer games.” and “I am not convinced with this treatment.”

The option that it would be a freely available treatment was regarded as an advantage by the majority. They report “Nice”; “Excellent”; “Good! Really good if it is for free.”; “Of course, because if it be online everyone can use it.” One participant was sceptic: “I’m not convinced, I don’t consider it a treatment and I have no comment.”

When asked how they would feel about *talking* about their traumatic experiences a large majority describe feelings of sadness, anger, pain and depression, e.g. “I feel sad, start day dreaming and experience a temporary shock”; “Depression and severe pain when recalling the painful events and the killing scenes we witnessed in our countries. It is sad.”; “Painful memories, I feel defeated and depressed when I remember them.”; “Feelings of sadness as I remember all the pain and suffering we have been through.” They describe reluctance to talk about it, e.g., “I don’t want to go through the past experiences again and sense the pain.” especially to a doctor, “It would be just ‘bla bla bla’ and not helpful.”; “I can’t talk to a doctor; prefer to talk to a friend.”; “Fear of sharing secrets and being exposed.”; “Maybe I can’t tell him all the things and all my memories and maybe some questions I can’t answer him.”.

Finally, attitudes toward taking part in a large-scale clinical trial testing this computer game-play intervention were generally positive: “of course I will take part if I have free time”; “I am willing to take part in this new experience. It might reduce my pain in an entertaining way”; “Of course, I’m ready to any research to help people”; “To great extent. Helping any person who has been through traumatic memories is duty”.
